# Hospital and Institutional News

**Published:** 1914-12-26

**Authors:** 


### HOSPITAL AND INSTITUTIONAL NEWS.

### CHRISTMAS FESTIVITIES IN WAR-TIME.

Now that Christmas is once again here, under unique circumstances so far at least as this generation is concerned, we hope, with the co-operation of our readers, once more also to publish next week a series of descriptive accounts. In case it should be thought that because, owing to the war, Christmas festivities have been cancelled in some hospitals, and considerably diminished in many others, these accounts will be less interesting or less desired by us, we beg to point out that quite the contrary is the case. We have, fortunately, not many opportunities of seeing how hospitals keep Christmas in war-time, and, therefore, peculiar interest attaches to the one occasion on which they are compelled to do so. Again, our accounts each year form a valuable chapter in hospital history, and so striking and exceptional an occasion must have its due at the hands of institutional writers. This year, more than usual, will it interest one institution to know what another has achieved in this respect. It may be that decorations, entertainments, and novelties generally will be more than usually impromptu in character, but, if so, they will, we are convinced, make all the better reading. We therefore remind our readers that all accounts, with or without photographs of interesting features, should reach us, if possible, not later than Tuesday morning, December 29, first post. If this rule be complied with they will be in time for insertion in The Hospital dated January 2. Preference, we may add, will be given to accounts from institutions which were *not* represented last year, and all, whether sanatorium, Poor-Law infirmary, cottage hospital, or, of course, general hospital, are eligible.

### EAST COAST HOSPITALS AND THE RAID.

The hospitals of Scarborough, West Hartlepool, and Whitby all appear to have been involved in the raid which five of Germany's fastest warships made on these unfortified pleasure and industrial towns on Wednesday last week. As far as information is to hand at the time of writing seventeen persons were killed at Scarborough, and twenty wounded people were received at the Scarborough Hospital and Dispensary, an institution, it will be recalled, of fifty beds, which was rebuilt in 1893. In the two boroughs of the Hartlepools nearly one hundred persons were killed, and several hundred have received injuries, particularly those in the old borough, which suffered much more severely than the newer districts of West Hartlepool. "A violent earthquake," wrote one correspondent, "could not have caused the same measure of ruin as this bombardment by heavy guns." On all hands the escape of the Hartlepools Hospital, in Friar Terrace, is described as miraculous, for institutions or buildings on three sides of it were all struck by shells. For instance, the site immediately behind the hospital is occupied by a school, which was struck, while the houses at the side, and also, we may note, in front of the hospital, were simultaneously damaged. A Red Cross flag was flying over the hospital, but the apparent want of discrimination in the shooting—some shots fell outside the town even—makes it uncertain whether this can be attributed to the accuracy of the German gunners. A Red Cross hospital near Londesborough Lodge was not so fortunate, for the buildings by the Crescent containing wounded soldiers were heavily bombarded. Londesborough Lodge, also turned into a Red Cross hospital, escaped, however. Possibly this was due to the fact that it was a low building above which several of the shells flew, but many windows were broken by the concussion.

### THE NEW PROFESSOR OF BIO-CHEMISTRY AT CAMBRIDGE.

Mr. Frederick Gowland Hopkins, of Trinity College, has been elected to the Professorship of Bio-Chemistry at Cambridge. Already a Fellow of his College, honorary Fellow of Emmanuel, and a Fellow of the Royal Society, Mr. Hopkins, who holds the University Readership in Chemical Physiology, is also a medical graduate. While continuing to hold the Readership in Chemical Physiology, in conjunction with the Professorship, to which no salary is attached, the new Professor is also a member of the Government Medical Research Committee, and one of the official analysts to the Home Office. He took the degree of M.B. with honours at London University in 1894, and became a Fellow of the Royal College of Physicians in 1914. He studied at Guy's Hospital, and was formerly Demonstrator of Physiology and Chemistry and first holder of the Gull Research Studentship at that institution.

### LIVERPOOL MERCHANTS' MOVABLE HOSPITAL.

Dr. Nathan Raw and Mr. T. C. Litler Jones, of Liverpool, are to be placed in charge of the movable temporary hospital which funds in Liverpool are providing for the prompt treatment of serious cases in the field. It appears that the merchants who have raised the money desire to achieve the above object by having their movable hospital constructed in sections, so that, it is claimed, it can be put up in twenty-four hours and pulled down and neatly packed for transport in forty-eight. Thus, provided the advance be not too rapid, it will be able to follow the movements of the troops and be within the nearest safe distance of the firing-line possible. It is to be, in short, a sort of travelling base hospital for serious cases which require immediate medical and surgical aid. It is interesting to note that the plan provides for no fewer than eight wards, on the pavilion scheme, and each of these is to contain twenty-six beds. The hospital as planned will cover 4,270 sq. yards. The nursing staff consists of forty, and the *personnel*, like the funds, are being found locally. Miss Whitson, lady superintendent of Brownlow Hill Infirmary, has been appointed matron. Up to the present £21,795 has been received, and in addition monthly subscriptions amounting to £750 have been promised. Lord Derby is the President of the committee responsible for the arrangements. It will be instructive to watch the success of the project, for, as the officers of the Royal Army Medical Corps who have been writing to The Hospital from the Front have shown, one of the chief difficulties has been, and is, to decide upon the safety for hospital purposes of any spot at all within the range of the firing-line. It is necessary to emphasise this point, since the movable hospital must at all costs escape from being shelled if it is to carry on the work for which it has been provided.

### KING'S COLLEGE HOSPITAL REMOVAL FUND.

One of the institutions to which the war has brought perhaps the greatest responsibility and anxiety is the new King's College Hospital on Denmark Hill. Immediately on the outbreak of war the greater part of the new institution was handed over to the military authorities for the use of the 4th London General Hospital, R.A.M.C. (T.). No fewer than 500 beds were placed at their disposal; and, in order to provide so much accommodation, the out-patient department was obliged to be closed. To meet the needs of patients thus inconvenienced temporary arrangements have been made for dealing with children and those cases which are sent by medical practitioners. In the meantime the number of wounded treated by the institution at one time averages 400 in the military wards, and in this number are included 120 Belgian soldiers. The result has been that money is urgently required to maintain the existing portion of the hospital, as well as to complete the building in accordance with the original plan. To build the remaining wards about £50,000 would be required, and the case for the hospital is, as the above summary shows, a threefold one. So large an institution is, of course, very costly to maintain, and among those hospitals who are about to reap the reward of increased public confidence for the way in which they have generously aided the nation since the war, King's, it may be hoped, will have a high place.

### THE NIGHT ATTENDANCE OF INSURED PATIENTS.

This question, always a thorny one in any form of contract practice, has been lately causing some discussion at the meetings of the West Riding Insurance Committee. Certain model rules, which the Medical Benefit Sub-Committee recommended for adoption, included a clause to the effect that an insured person "shall not summon the practitioner to visit him between the hours of 8 p.m. and 8 a.m. except in cases of serious emergency." This clause it was proposed to eliminate. One speaker stated that the sub-committee had aimed at making the rules less stringent and less oppressive for the insured person. A medical man, however, who was present argued that an effort was being made to take advantage of the medical profession during hours in which it was only reasonable that they should be, generally speaking, free. The amendment was eventually carried. Those experienced in contract practice realise more than anyone else perhaps how little the letter of regulations has effect on the end at which they aim, if the will and hearty co-operation of all parties subject to them are not present. That is really the crux of all questions of which the above is only a type.

### AN ARCHITECT AS HOSPITAL SECRETARY.

The committee of Worthing Hospital has appointed Mr. Arthur H. Tucker to the office of Secretary to the hospital in succession to Captain Montague J. Prentice. The new Secretary, it is interesting to note, is by profession an architect and surveyor, and were the Worthing Hospital a larger institution than it is it would be worth while enlarging upon the effect of combining professional architectural knowledge with a post as hospital manager. The new hospital, we may recall, was established in 1881, and consists at present of twenty-one beds and eight cots. Two years ago the new out-patient department was opened, which formed the town's memorial to King Edward.

### THE SHORTAGE OF MEDICAL OFFICERS.

Owing to the difficulty of obtaining medical officers the Hammersmith Guardians have decided to ask the Local Government Board to sanction the appointment as junior medical officer of a gentleman who has passed a portion only of his final examination. This request would appear to have been made without any attempt on the part of the Guardians to increase the present inadequate salary they pay in order to obtain a fully qualified man. We hope that both in this instance and in the case of the Lambeth Guardians, to which we have previously referred, the Local Government Board will take up a firm attitude, and will refuse to permit any deterioration of the medical staffs in the Poor-Law infirmaries until better evidence is forthcoming than has hitherto appeared that the shortage of medical men is such that a reasonable salary fails to obtain fully qualified men or women. The root of the evil, as we have pointed out before, is the mistaken parsimony of the Guardians and the Local Government Board in the past. The result has been so to disgust the medical profession with the indoor Poor-Law Medical Service that the first effect of the increased demand of recent years for medical men has been to diminish, and finally to cut off, the supply of candidates for the service. A reconstitution of the service so as to make it attractive to medical men cannot be organised in a hurry, but it is essential that every possible means of obtaining properly qualified men should be exhausted before recourse is had to the desperate expedient of introducing unqualified men. The Poor-Law infirmary is in a very different position from the voluntary hospital. The smallness of the staff causes a large amount of responsible work to be thrown upon even the junior medical officer, and it is impossible to give him that amount of supervision by his senior colleagues which is feasible in the case of the voluntary hospital.

### "DUMMY" TEATS AND RUBBER TOYS.

The "dummy" teat has often been condemned as one of the alleged causes of adenoids, but this article, which is so familiar in households where there are babies, has now been adversely criticised from another point of view. The United States Government has issued an official publication of the Department of Hygiene, in which are recorded the results of the examination of a number of rubber teats, teething-rings, and rubber toys, and it is evident that many of these articles which are on the market consist of rubber of decidedly objectionable character. Of thirteen varieties of rubber teats examined all contained iron and aluminium in appreciable quantities, while in nine of them antimony was found. The presence of antimony suggests that rubber scrap or recovered rubber is used in compounding the rubber from which the teats are made. Obviously the use of antimony rubber for the manufacture of rubber teats is highly undesirable, and it is not surprising that the Department of Hygiene condemns its use for this purpose, and points out that these teats should be made of a good grade of black rubber free from rubber scrap and recovered rubber. The same recommendation applies to rubber teething-rings and rubber toys.

### ADEQUATE SUBSCRIPTIONS TO HOSPITALS.

The question as to what constitutes an adequate subscription to a hospital by a Board of Guardians has been raised by the St. John's Hospital for Diseases of the Skin with reference to the patients treated there from the institutions of the Lambeth Board of Guardians. Within a comparatively short period three cases have been treated at the hospital without payment, save for an annual subscription of one guinea made by the Guardians. The hospital authorities thought this was inadequate, and asked that definite arrangements might be made. The board, however, have come to the conclusion that it will be more desirable to continue the payment of an annual subscription, which is in future to be increased from one to three guineas. It is somewhat doubtful whether this augmented subscription will satisfy the hospital authorities, who are certainly not called upon to be public benefactors at the cost of their own finances where a public authority has means by which to pay adequately for the treatment of its poor.

### THE WOMEN'S VOLUNTARY HOSPITALS BRIGADE.

The response we have received to the proposal to form a brigade of workers for the voluntary hospitals, which we explained in our issue of November 28, pp. 185-6, and December 5, p. 215, proves that the idea commends itself to large numbers of women up and down the country, who recognise the great work that is being done by our voluntary hospitals for the sick and wounded in the war. This response, therefore, encourages us to proceed, and steps will now be taken to organise the Brigade so that it may be in active working early in the New Year. As soon as all arrangements are complete those intending members who have communicated with us will be notified. Meanwhile we suggest that they should direct their energies to an active canvass of their friends, who should be asked either to undertake to raise £1 each per annum for the period of the war, or to promise to contribute, when called upon, to the League of Mercy through the Women's Voluntary Hospitals Brigade small sums of money from 1s. upwards. All money so collected will be paid to the hospitals and will thus be of direct benefit to the sick and wounded in the war.

### THE POWER OF THE PENCE.

Those interested in the aim and intention of the Women's Voluntary Hospitals Brigade referred to above should study the report of the annual meeting of the Presidents of the League of Mercy, reported on p. 294 of this issue. From the Treasurer's speech they will see that during 1914 the amount collected by the League of Mercy workers is over £18,000, nearly the whole of this sum being raised by small subscriptions. Turning further to the Honorary Secretary's speech at the same meeting, they will read that in two districts of London over 8,000 separate penny collections were made in 1914 on behalf of the League of Mercy. Another instance of the value of the small subscriber is to be found in the Borough of Leicester, where, at St. Margaret's Works, the large sum of £914 6s. 9d. has been raised from workpeoples' contributions during 1914 for various institutions and funds. This large sum is the result of voluntary gifts, and is quite independent of the firm's charitable contributions.

### FINANCIAL STRAITS OF YORK COUNTY HOSPITAL.

A serious position of affairs was complained of at the recent quarterly court of the York County Hospital. The year began, the Secretary stated, with an adverse balance of £2,000. At the previous court Mr. Wilfrid Thompson, who succeeded the Dean of York as chairman of the house committee in June, remarked that a sub-committee had been appointed to canvass for new and increased subscriptions, but owing to the war the scheme has fallen into abeyance. The expenses exceed the income by about £1,000, which deficiency the committee desire to see met by annual subscriptions. "No hospital in the kingdom of the same size," the speaker added, "had so small a revenue from this source." In a remarkable speech the Dean of York attributed the hospital's difficulty in raising money at the present time to the recreation voluntarily provided by the citizens of York for the soldiers quartered in the city. "The Government," he is reported to have said, "provided these soldiers with board and lodging, but did nothing for their recreation. The consequence was that the provision of recreation made considerable demands on the people of York, which meant that they had less to give to local charities. He thought the provision of recreation might very well have been made a national charge." To avoid a great debt being accumulated on the hospital he suggested that they should draw upon the capital. If they did not do so, he suggested the only course open to them was to cut their coat according to the size of the cloth. At this point, we are glad to observe, someone had the sense to protest, and a voice was heard saying, "Get more cloth," which is the obvious alternative to the above policy of vacillation, want of energy, and weakness.

### ANOTHER HOSPITAL RUNNING AWAY.

As we pointed out in our issue of December 12, the committee of "every voluntary hospital which tends the wounded should realise that they have at present a ground of appeal that gives them the first claim upon all monetary gifts, as the public recognise." We then went on to criticise the policy pursued at Portsmouth by which, just as York Hospital is doing, "the committee bolted from their entrenchments as the first claimant" on the appearance of a variety of competing war funds. "Could ineptitude and ill-judgment," we added, "join hands with less reason and more discredit to the hospital authorities than this position reveals?" We fear that the same remark applies to the York County Hospital's authorities, for again we notice a tendency immediately to abandon all effort on the smallest sign of there being any difficulty to be overcome. At this season we are loth to press criticism, but we must remind all active minds interested in the welfare of this institution that a vigorous policy is always the best policy, and that, to administrators who understand their business and are awake to their opportunity, the treatment of wounded soldiers has given a force to any appeal that they press which is the unique opportunity of a generation. To run away, to give up, to withdraw the proposed scheme for raising new subscriptions now, when the hospitals' work for the nation is in every memory, is daily chronicled, is the talk of every town—while there is plenty of money still for charities which have proved their right to it—is a collapse of initiative and good sense which augurs ill for the future welfare of the institution concerned in it.

### MR. LLOYD GEORGE AND THE RED CROSS.

The Chancellor of the Exchequer has contributed a lengthy article to the Christmas Number of the *Methodist Times*, in which he gives some of his experiences on his recent visit to the battlefields of France. After relating an incident in which a French General, with whom the Chancellor was conversing, approached a wounded Prussian soldier and told him that he would be treated in hospital "exactly like one of our own men," to which the Prussian replied that equally kind treatment was accorded to the French wounded, Mr. Lloyd George expresses his wonder at the contrast between this incident and the artillery duel which was proceeding at the same time. "I marvelled," he adds, "that this exhibition of good will amongst men who were sworn foes should be possible amid such surroundings, until my eyes happened to wander down a lane, where I saw a long row of waggons each marked with a great red cross. Then I knew who had taught these brave men the lesson of humanity that will gradually, surely, overthrow the reign of hate." It is pleasing to set this personal experience beside those stories of the violation of the Red Cross which are indiscriminatingly urged against the combatants in all wars.

### THE METROPOLITAN HOSPITAL SUNDAY FUND.

As we go to press, one day earlier than usual this week on account of the Christmas holidays, the annual meeting of constituents of this Fund is being held. The business of the meeting is to elect new members of the Council, to re-elect retiring members who may be eligible, and to receive the report of the Council. This records a gratifying result of the year's collection, namely, a total of £65,400, being an increase of £409 compared with last year. The collections in the various places of worship resulted in a sum of £28,700, being £266 more than in 1913. The Council report that the appeals arising out of the disaster to the *Empress of Ireland*, which occurred just before last Hospital Sunday, probably affected the giving power of the public in some measure, and but for this disaster it is reasonable to suppose that this year's collection would have shown an even larger increase than it does on last year's total, despite the outbreak of war and the consequent necessity thought to be incumbent on some places of worship, where the Hospital Sunday collections had not already been made, to forego the collection for this year.

### THE ST. PANCRAS DISPENSARY SCHEME.

The London Insurance Committee have approved the scheme submitted by the St. Pancras Borough Council for the treatment of insured persons suffering from tuberculosis. The scheme provides for (1) the maintenance of two dispensaries, both of which are provided by voluntary authorities—one at University College Hospital and the other at the St. Pancras Dispensary; (2) the establishment of a branch dispensary in North St. Pancras; (3) the appointment on each dispensary committee of a representative of the local medical practitioners who are on the panel; and (4) payment to be made at the rate of £200 and £584 per annum in respect of the treatment of non-insured persons at the University College Hospital and the St. Pancras Dispensary respectively, and in respect of insured persons of a sum bearing the same proportion to these amounts as the number of attendances made by insured persons bears to the number of attendances made by non-insured persons.

### FRENCH RED CROSS SOCIETY.

The London Branch of the French Red Cross Society has been reorganised, and in future this branch, the headquarters of which are at 25 Knightsbridge, S.W., will be the only representative in London of the Union of the three Red Cross Societies established in France. Honorary and Executive Committees have been formed, among the members of the former being H.M. Queen Alexandra and the French Ambassador. The Vicomtesse de la Panouse is the president of the Executive Committee, and the honorary secretary is Mr. P. A. Wilkins.

### KING GEORGE HOSPITAL.

THE committee to carry out the arrangements for equipping and opening this hospital, which has been appointed by the Joint Committee of the British Red Cross Society and the Order of St. John, consists of the following gentlemen: The Hon. Arthur Stanley, M.V.O., M.P. (chairman of Joint Committee), Colonel Sir Herbert Perrott, Bart., C.B. (vice-chairman of Joint Committee), Sir Frederick Treves, Bart., G.C.V.O., Mr. Edmund Owen, F.R.C.S., the Hon. W. H. Goschen, and Sir Robert Hudson, being three representatives of each of the two Societies, with Sir Rowland Bailey, C.B. (for many years head of His Majesty's Stationery Office). Mr. E. W. Morris, the secretary of the London Hospital, will act as hon. secretary of the hospital, and Mr. Oakley Williams, who, we understand, has done a considerable amount of work at the London Hospital, as secretary. Miss Davies, the late matron of St. Mary's Hospital, has been appointed matron.

### CASUALTIES AMONG GERMAN ARMY DOCTORS

THE Berlin correspondent of the official organ of the American Medical Association, writing under date October 28, 1914, states that nearly one hundred out of about 9,000 surgeons at that date with the German Armies have fallen, and thirty-seven have been wounded, besides thirteen missing and three taken prisoners. He compares these figures with the casualties in the German Medical Service during the whole of the Franco-Prussian war of 1870-1, when of 4,062 surgeons on the field of battle on the German side only nine fell and two later succumbed to their wounds. The reason for the disproportionately large number of casualties among the Army doctors is, he says, that owing to the great distance at which the present firearms—not only of the artillery, but even of the infantry—are fired it is inevitable that dressing stations should be placed too near the firing-line and reached by missiles. Further, in view of the great distances at which the opposing forces so often stand, the Red Cross cannot always be recognised. With regard to the nature of the wounds of the German soldiers and the general health of the German Army, he says that the wounds heal with surprising quickness and with proportionately small functional disturbance. There is, however, a large number of tetanus cases. Otherwise relatively little disease has been reported, although both in the East and in the West there have been a few hundred cases of typhoid and dysentery, but these are quite sporadic, and not in the nature of an epidemic.

### DEATH OF A DISTINGUISHED BELGIAN SCIENTIST.

WITH unexpected suddenness the famous scientist, Professor P. L. A. van Gehucten, of Louvain University, died at the Research Hospital, Cambridge, last week, having recently undergone a surgical operation from which he was recovering satisfactorily until he suddenly became worse and died the same day. Professor van Gehucten was one of the most eminent members of the Belgian Royal Academy of Medicine. He taught at Louvain anatomy and neuropathology, having made a special study of the nervous system. The works which he had published upon his subject had gained for him a world-wide reputation. The late professor was adored by his students for his accurate and clear teaching, and not less by his colleagues for his amiable, persuasive, and inspiring eloquence. He and his family had found refuge in Cambridge in consequence of their homes in Louvain and Middelkerke being destroyed, and the professor had taken up residence at the Research Hospital for the purpose of instituting some new experiments, which have been abruptly ended by his untimely death.

### A SURCHARGE FOR ASSISTANCE AT OPERATIONS.

THE official audits of the accounts of Boards of Guardians often produce results which suggest that the regulations of the Local Government Board governing them are badly in need of revision. A surprising instance, but one which is not unique, occurred recently in the case of the Lexden and Winstree Guardians. Two members of the Board were surcharged because they had sanctioned, without having received the consent of the Local Government Board, the payment to the medical officer of the workhouse of the expenses he had incurred in obtaining assistance for the performance of an operation on an inmate. The auditors' decision is no doubt technically correct, but this does not render it any the less absurd. Operations cannot as a rule wait upon the observance of official formalities. The surcharge will no doubt be remitted upon appeal, but the whole procedure is obviously wrong. A surcharge is meant to act as a deterrent from the repetition of an offence. In this case there is no suggestion of an offence; on the contrary, the breach of any formalities involving unnecessary delay is praiseworthy. The Local Government Board should at once take steps to amend their regulations so as to prevent their auditors interfering in any way with the Guardians in the performance of their duties towards the sick for whom they are responsible.

### ST. KATHARINE'S HOSPITAL AGAIN.

THE Local Government Committee of the London County Council have expressed their regret that in the recent leasing of premises for the purposes of St. Katharine's College the claims of the district contiguous to and in the immediate neighbourhood of the site of the ancient foundation have not been adequately recognised, and their hope that in connection with any extension of the work or the establishment of a permanent institution therefor such claims shall be met. When the new rules for the management of St. Katharine's Hospital were framed it was stated that there was to be established, "as near as may be to the site of the ancient foundation," a college for the provision of duly qualified resident health visitors, to devote themselves mainly to maternity and health visiting work in the poorer districts of East London. It now appears that the premises which have been taken temporarily for the purposes of the college at Poplar, where four health visitors have already commenced work, are about two and three-quarter miles from the site of the ancient hospital, and the action of the Chapter, in the view of the committee, does not give full effect to the intention of the new rules. While they do not suggest that the institution at Poplar is unnecessary, they regret that the institution is so far away from the site of the ancient foundation. The Stepney Borough Council also intends to remonstrate with the authorities on the subject.

### THE HOSPITAL SATURDAY FUND.

At the next meeting of the Board of Delegates of the Hospital Saturday Fund the Executive Committee will report that Sir Chas. Johnston, the present Lord Mayor of the City of London, has consented to act as President of the Fund during his year of office. The contributions to the Fund from the workshops and business houses, etc., of London to Saturday, December 19, amounted to £20,509, as against £20,393 at the same date last year. As showing the effect which the war has had upon the collections, the receipts on July 31 were £1,040 in advance of the corresponding period of 1913, while on December 5 they were only £451 ahead. The special efforts made by the local committees on Hospital Saturday, October 10, were generally successful. Those at the cinematograph theatres, the Collection Committee report, were particularly satisfactory. The Fund has sustained the loss of one of its oldest honorary collectors, Mr. Wm. Mason, who is reported to have gone down with H.M.S. *Hawke* on October 15.

### THE NEW MATRON AT COVENTRY.

Once more the success of members of the various staffs of King Edward VII.'s Hospital, Cardiff, in attaining to important positions is shown by the appointment of its assistant matron, Miss S. Hutchinson, as matron of the Coventry and Warwickshire Hospital. Miss Hutchinson has been at Cardiff three and a half years, and will be greatly missed for her geniality and general ability. After training at the Manchester Royal Infirmary she became night sister at the County Hospital, Ryde. Her next position was that of senior sister and deputy matron at the Miller General Hospital for South-East London, and prior to going to Cardiff she was senior assistant deputy matron at the Princess Alice Hospital, Eastbourne.

### FOREIGN LANGUAGES MADE EASY.

Englishmen are supposed to be bad linguists, but this national defect, in so far as it exists, is, like many other qualities, due more than anything else to our geographical position. An island, even a short distance from the Continent and in spite of modern means of quick transit, is inevitably much cut off from intercourse with foreigners. Indeed, that strip of water appears to have a definite hypnotic influence, so that while people think next to nothing of travelling from London to Edinburgh, they imagine a great many impossible difficulties to stand in the way of a journey from London to Paris, which is about the same distance. Unlike Frenchmen, Germans, and Italians, we rarely hear any other tongue than our own, and thus we trouble very little to learn foreign languages—a characteristic piece of laziness which has caused us much unnecessary difficulties since the war began. Doctors and nurses have been much handicapped by knowing no French and no German, and therefore those active spirits who are anxious to repair this grave defect in their education will appreciate the little, inexpensive booklets, by Captain J. S. Keyworth, "Easy French" and "Easy German," which are obtainable at 56 Church Road, Hove. To the several conversational volumes issued with a similar object the two mentioned are useful adjuncts, and we trust that all those whom experience has already taught the disadvantage of their ignorance, will take advantage of their cheapness to gain at least a rudimentary knowledge of both these languages.

### THIS WEEK'S DRUG MARKET.

As is usual at this time of the year, business in the drug market is almost at a standstill, and until the process of stocktaking is over, no improvement is to be expected. Early in the New Year, however, business may be expected to begin again in real earnest and to be more active than it has been for some time past. Buyers have now become accustomed to paying the advanced prices due to the war and realise that there is not much advantage to be obtained by delaying purchases; while the decrease in the consumption of drugs—if there has been any decrease—has only been slight. There has been a further advance in the price of cod-liver oil, the increase this time being fairly substantial; the quantity available both here and in Norway is no doubt large, but the unknown factor is the condition under which next season's fishing operations will be conducted. There is at least the probability of danger from floating mines, and it is possibly the prospect of such a contingency that has helped the upward movement of prices. Opium, morphine, and codeine are practically unchanged, but prices are well maintained. Carbolic acid still has a tendency towards higher prices. Bromides are unaltered. Citric acid, tartaric acid, cream of tartar, essence of lemon, oil of orange, and oil of bergamot have a downward tendency in price, although this is not very pronounced. There has been little change in the quotations for synthetic drugs, and no doubt the decrease in demand, which is largely due to the "war prices," is preventing a further substantial increase in value.

### TO OUR READERS.

Contributions are specially invited from any of our readers to these columns. They should deal with topical subjects and news. They must be authenticated for the information of the Editor only. The minimum payment if published is 5s. There is no hard-and-fast rule as to space, but notes of about twenty lines in length are preferred.

